# Tumor-Associated Macrophages Contribute to Tumor Progression in Ovarian Cancer

**DOI:** 10.3389/fonc.2014.00137

**Published:** 2014-06-06

**Authors:** Emily K. Colvin

**Affiliations:** ^1^Bill Walsh Translational Cancer Research Laboratory, Kolling Institute of Medical Research, Royal North Shore Hospital, University of Sydney, St. Leonards, NSW, Australia

**Keywords:** ovarian cancer, tumor microenvironment, tumor-associated macrophages, macrophages in ovarian cancer

## Abstract

Ovarian cancer is the leading cause of death in women with gynecological malignancy and improvements in current treatments are needed. As with many other solid cancers, the ovarian tumor microenvironment is emerging as a key player in tumor progression and a potential therapeutic target. The tumor microenvironment contains several non-malignant cell types that are known to contribute to tumor progression and metastasis. Included in this population of non-malignant cells are several different types of immune cells, of which tumor-associated macrophages (TAMs) are the most abundant. An increasing amount of evidence is emerging to suggest that TAMs display a unique activation profile in ovarian tumors and are able to create an immunosuppressive microenvironment, allowing tumors to evade immune detection and promoting tumor progression. Therefore, an increased understanding of how these immune cells interact with tumor cells and the microenvironment will greatly benefit the development of more effective immunotherapies to treat ovarian cancer. This review focuses on the role of TAMs in the ovarian tumor microenvironment and how they promote tumor progression.

## Introduction

Epithelial ovarian cancer (EOC) represents the leading cause of cancer mortality in women with gynecological malignancy (1). The overall 5-year survival rate for EOC patients is approximately 40%, although in the majority of patients diagnosed with advanced disease, the survival rate is significantly less (2, 3). Current standard treatment for EOC involves surgical debulking followed by platinum-based chemotherapy. Although initial response to chemotherapy is high, recurrence of chemoresistant disease is common and is a major contributor to the poor prognosis of EOC.

While much effort has gone into uncovering the genetic drivers responsible for EOC initiation and progression, the tumor microenvironment is now increasingly recognized to play an important role in EOC. The tumor microenvironment consists of several different cell types that interact with tumor cells, and with each other, to influence tumor initiation, growth, and metastasis. Immune cells represent a major component of the tumor microenvironment and allow tumor cells to evade immune destruction.

Evidence suggests that ovarian tumors, like many solid tumors, are immunogenic, containing tumor infiltrating lymphocytes that indicate an interaction between tumor cells and the host’s immune system. In a seminal paper published by Zhang et al., the presence of CD3+ infiltrating T cells in tumors was shown to significantly increase long term survival in patients with advanced ovarian cancer (4). Five-year overall survival rates were 38% in patients that contained infiltrating T cells in their tumors compared to <5% in patients that contained no T cells. Furthermore, multivariate analysis showed that the presence of intratumoral T cells was an independent prognostic factor. Since then, several studies have confirmed the positive association between the presence of tumor infiltrating T cells and patient survival (5–8). The influence of intratumoral T cells on patient outcome indicates the immune system may play an antitumor role in ovarian cancer; however spontaneous regression of tumors through immune destruction is rare. In addition to cytotoxic T cells that display antitumor characteristics, ovarian tumors contain a plethora of other immune cell types that create an immunosuppressive tumor microenvironment. These include regulatory T cells (T_regs_), dendritic cells, myeloid-derived suppressor cells, and tumor-associated macrophages (TAMs). TAMs represent the most abundant immune cell type in the ovarian tumor microenvironment and play several roles in promoting tumor progression. While all of these cell types have been shown to play an important role in ovarian cancer, some of which have been reviewed elsewhere (9, 10), this review focuses on the characteristics of ovarian TAMs and their role in ovarian cancer.

## Tumor-Associated Macrophages

Macrophages are phagocytic cells of the immune system that are derived from circulating monocytic precursors, which extravasate into tissues and differentiate in response to local signals. They represent a heterogeneous population of cells that can function to stimulate the immune system or to suppress it. This heterogeneity has been simplified to group macrophages broadly as either “classically activated” or “alternatively activated” (11). Classically activated macrophages, also known as M1-polarized macrophages, are activated by cytokines such as interferon-γ (IFNγ) and produce pro-inflammatory and immunostimulatory cytokines (e.g., IL-12 and IL-23), and are involved in Th1 responses to infection. In contrast, alternatively activated, or M2-polarized macrophages, are activated by Th2 cytokines (e.g., IL-4, IL-10, and IL-13) are immunosuppressive, and are involved in scavenging cellular debris and tissue repair. In general, TAMs are thought to more closely resemble the M2-polarized phenotype (12). The function of TAMs has been extensively studied in many cancer types and in addition to playing an immunosuppressive role in the tumor microenvironment, TAMs have been shown to promote tumor invasion, growth, angiogenesis, and metastasis (13, 14). Given the variety of roles that TAMs play in tumor progression, it is not surprising that their presence in many tumor types is often associated with poor prognosis (15–17).

## Recruitment and Characteristics of TAMs in Ovarian Cancer

Tumor-associated macrophages represent the most abundant infiltrating immune population in human ovarian tumors and ascites (18). Ovarian tumors recruit circulating monocytes and induce differentiation into TAMs via expression of factors such as CCL2, also known as monocyte chemotactic protein-1 (MCP-1), and macrophage colony stimulating factor-1 (M-CSF or CSF-1). CCL2 is overexpressed in ovarian tumor cells and cell lines, but not in TAMs (19). Interestingly, expression of its receptor, CCR2 is defective in TAMs derived from ovarian cancer patients (20). This may reflect a mechanism by which ovarian tumors retain recruited macrophages in the tumor microenvironment. CSF-1 is a cytokine considered to induce differentiation of macrophages to an M2 phenotype (12) and is overexpressed in human ovarian cancers (21, 22). Expression of CSF-1 is higher in malignant ovarian tumors compared to borderline and benign tumors (23).

Initial studies characterizing TAMs in ovarian cancer demonstrate that TAMs most closely resemble M2-polarized macrophages and express M2 markers such as CD163, CD204, CD206 (Mannose Receptor), and IL-10 (23–26). Ovarian TAMs also express the immunosuppressive chemokines CCL18, which is found in high levels in ascites from ovarian cancer patients (27) and CCL22 (28). More recently, genome-wide expression profiling has been used to investigate the polarization of TAMs in patients with high-grade serous ovarian cancer. The transcriptome of 17 human ovarian TAM samples was compared to non-polarized (M0) macrophages and identified differential expression of 1275 genes. Further analysis of these genes revealed that ovarian TAMs display a mixed-polarization phenotype. TAMs displayed upregulated expression of typical M2 markers such as CD163 and IL-10, while other M2 markers were downregulated. Similarly, some M1 markers were upregulated in ovarian TAMs, such as CD86 and TNF. This mixed-polarization phenotype has also been described in other tumor types (29–32), and suggests that TAMs most closely resemble macrophages involved in developmental processes.

## TAMs and Prognosis in Ovarian Cancer

Studies investigating the presence of TAMs in ovarian cancer demonstrate a significant increase in the number of TAMs in malignant ovarian tumors compared to benign and borderline tumors (23, 33, 34). However, their presence as determined by staining for the macrophage marker CD68 does not influence patient outcome (33, 35–37). Expression of specific M2-associated markers in ovarian cancer indicates that certain subsets of ovarian cancer TAMs can indeed predict patient prognosis. In addition to CD68+ cells, Lan et al. also analyzed 110 advanced stage ovarian cancers for the M2 marker CD163 and demonstrated that both progression free survival and overall survival were significantly reduced in patients with high numbers of CD163+ cells (37). Serum levels of CD163 have also been shown to predict poor prognosis in patients with ovarian cancer (38). Similarly, another M2-associated marker was associated with poor prognosis in ovarian cancer. While absolute densities of CD206+ cells were not prognostic, a high CD206/CD68 ratio was strongly associated with worse progression free survival, and there was also a trend toward poorer overall survival (35). Recent studies examining markers of both M1 (HLA-DR, iNOS) and M2-polarization (CD163, VEGF) in ovarian cancer patients have demonstrated that an increased M1/M2 ratio was associated with improved patient survival (39, 40). One study quantified the M1/M2 ratio in the tumor and the stroma and found that only the M1/M2 ratio of overall tumor macrophages or macrophages present intratumorally were prognostic, the M1/M2 ratio in tumor stroma was not predictive of improved survival (39), indicating that macrophages infiltrating tumor cells may play a more important role in tumor progression. Finally, expression of B7-H4 on the surface of ovarian TAMs, but not expression in ovarian tumor cells, was associated with reduced survival and the number of B7-H4+ macrophages was significantly increased in advanced disease (41). These studies demonstrate that while total numbers of CD68+ macrophages present in ovarian tumors do not influence patient outcome, there is strong evidence for specific subsets of TAMs as prognostic factors in ovarian cancer.

In addition to surface markers present on TAMs, cytokines that are important in TAM function are also elevated in human ovarian cancers and associated with reduced survival. IL-6, which is present at high level in ovarian cancer ascites and associated with the generation of TAMs (42), is associated poor prognosis and chemoresistance (43–45). Similarly, IL-10, produced by TAMs, is increased in ovarian cancer and correlated with higher tumor grade and poor patient outcome (45–48). High levels of CSF-1 have also been shown to be a poor prognostic factor in ovarian cancer, when expressed in the tumor epithelium (21). Expression of specific markers of ovarian TAMs, as well as cytokines that are important in TAM function and recruitment acting as prognostic factors in human ovarian cancer provide strong support for the function of TAMs in ovarian cancer progression.

## Interactions between TAMs and Ovarian Tumor Cells

**Figure 1:**
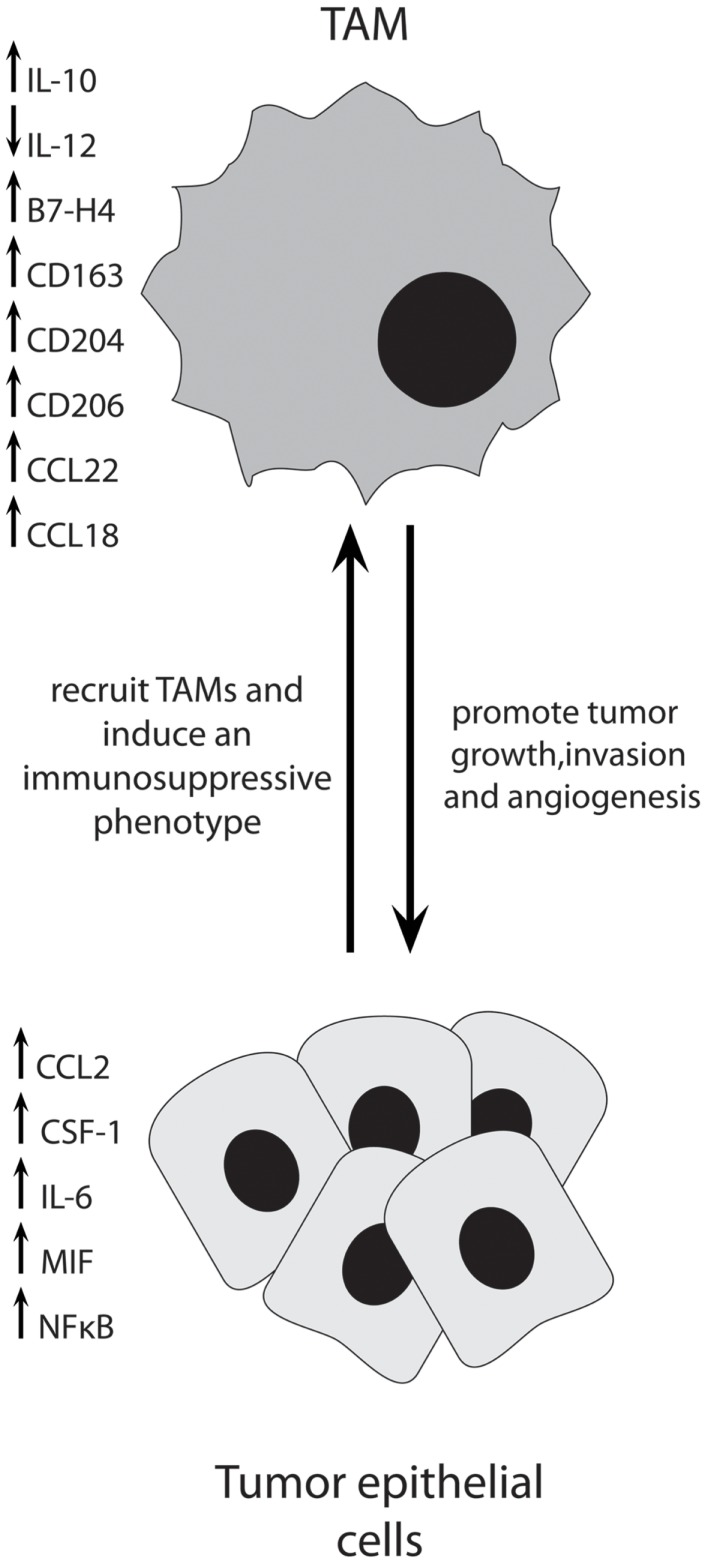
**Ovarian cancer cells produce multiple factors to recruit immunosuppressive TAMs into the tumor, where they act to promote tumor progression through multiple mechanisms**.

Ovarian cancer cells produce a variety of factors that influence TAM function and vice versa. Co-culture experiments using ovarian cancer cell lines and macrophages have revealed much about the interactions between these two cell types. Ovarian cancer cells have been shown to recruit and induce differentiation of macrophages that have tumor-promoting functions. Figure 1 depicts some of the important interactions between TAMs and ovarian cancer cells.

Following co-culture with ovarian cancer cells, macrophages develop a cell-surface phenotype similar to TAMs isolated from human ovarian tumors and a significant increase in genes such as *CCL2, CCL22, TNF*α, *TGFβ1*, and *VEGF* (49). In addition, co-culture with cancer cells upregulated the M2-associated Mannose Receptor (CD206) and Scavenger Receptor-A (SR-A, CD204), which was not seen when macrophages were co-cultured with normal ovarian surface epithelial cells (49). The induction of SR-A on macrophages was dependent on the presence of TNFα. Expression of macrophage migratory inhibitory factor (MIF) and extracellular matrix metalloprotease inhibitory factor (EMMPRIN) by ovarian cancer cells induces an increase of MMP secretion by macrophages (50), supporting a role for TAMs in tumor cell invasion and angiogenesis. Downregulation of MIF in ovarian cancer cells led to a decrease in the production of cytokines important in TAM recruitment such as CCL2 and CCL22 *in vitro* and an increase in survival and decrease in ascites *in vivo* (51). Furthermore, inhibition of MIF in ovarian tumor-bearing mice resulted in a decrease in proliferation, an increase in tumor cell apoptosis, and a decrease in the expression of angiogenic factors such as VEGF by tumors. Importantly, there was also a significant decrease in macrophage infiltration in the ascites, as well as a decrease in IL-6 and TNFα, and an increase in M1-associated IL-12 (51). Ovarian cancer cell lines and tumor biopsies have been shown to express elevated levels of IL-6, TNF, CXCL12, and its receptor CXCR4, and expression of these is co-regulated (52, 53). Decreasing the levels of all of these cytokines and chemokines in ovarian cancer cells was achieved by knocking down CXCR4 (52). When injected into mice, CXCR4 knock-down cells produced a decrease in tumor growth and an increase in survival. A significant decrease in the number of macrophages in tumors was also seen. Similar results were achieved when mice were treated with an anti-TNF antibody (52). These studies demonstrate that ovarian tumor cells employ several methods to recruit and induce TAMs to an immunosuppressive phenotype in the tumor microenvironment.

Due to the association between increased levels of IL-6 in ovarian cancer patients and the development of chemoresistance (44), Dijkgraaf et al. investigated the effect of platinum-based chemotherapy on the differentiation of macrophages *in vitro*. Treatment of ovarian cancer cell lines with cisplatin or carboplatin led to an increased ability of some cell lines to induce differentiation of monocytes to an M2-like phenotype (54). The underlying mechanism behind this was due to chemotherapy-induced activation of the NFκB pathway, which resulted in an increase in IL-6 and prostaglandin E_2_ by cancer cells that promoted M2-polarization of macrophages. These results indicate that therapeutically inhibiting this effect, for example, by blocking the IL-6 receptor may increase the antitumor effects of platinum-based chemotherapy.

Ovarian tumor cells are a heterogeneous population in which Alvero et al. have identified two distinct subpopulations that have different stemness, inflammatory, and cytokine profiles (55–57). Interestingly, these two populations of cells were found to have unique effects on the differentiation of macrophages (58). Monocytes cultured in Type I EOC cell (CD44+/MyD88+, cancer stem cells) conditioned media demonstrated increased levels of scavenger receptors and cytokines important in tissue repair such as CCL5, whereas those cultures in Type II cell conditioned media demonstrated increases in IL-10, IL-8, and G-CSF and are more likely to play an immunosuppressive role in the tumor microenvironment (58). Nonetheless, both tumor cell populations differentiated monocytes into macrophages with tumor supportive, rather than antitumor properties, and these results add another level of complexity to interactions between ovarian tumor cells and their microenvironment.

Tumor-associated macrophages have been found to promote the invasiveness of ovarian tumor cells through multiple mechanisms. Co-culture of macrophages with human ovarian cancer cell lines increases the invasiveness of tumor cells through TNFα-dependent activation of JNK and NFκB signaling pathways (50). Inhibition of IKKβ, a major activator of NFκB signaling, in ovarian TAMs prevented tumor cell invasion as well as decreased TAM production of M2 immunosuppressive cytokines and increased production of M1-associated IL-12 and NOS2 (59). Adoptive transfer of IKKβ-targeted TAMs into ovarian tumor-bearing mice resulted in a significant decrease in tumor burden and a switch to an antitumor TAM profile (59). The ability for TAMs to promote tumor cell invasion is also dependent on expression of SR-A. SR-A^−/−^ macrophages displayed a reduced ability to promote invasion of ovarian cancer cells *in vitro* and slowed tumor progression *in vivo* (60). Importantly, this study also demonstrated that targeting SR-A therapeutically with a small molecule inhibitor can prevent tumor progression *in vivo*. Another study also demonstrated the important role macrophages play in ovarian tumor progression by chemically depleting macrophages *in vivo* with clodronate, which dramatically decreased tumor dissemination and the development of ascites in mice injected intraperitoneally with ovarian cancer cells, potentially due to a decrease in VEGF production (61). These studies demonstrate that TAMs promote ovarian tumor progression by employing several different strategies.

Tumor-associated macrophages foster an immunosuppressive microenvironment to promote the survival of tumor cells. Monocytes and macrophages derived from peripheral blood and ascites of ovarian cancer patients were found to be increased in number and to display a less differentiated phenotype compared to cells derived from healthy donors (62). They were also shown to have impaired antitumor activity due to defective cytotoxicity and phagocytic abilities. Another mechanism by which TAMs promote immunosuppression is via secretion of CCL22, which mediates T_reg_ cell trafficking to the tumor (28). B7-H4, which is expressed on the surface of ovarian TAMs, also contributes to immunosuppression in the tumor microenvironment. B7-H4 expression is induced by IL-6 and IL-10 and selectively blocking B7-H4 in macrophages significantly increased T-cell proliferation, whereas ectopic expression of B7-H4 in macrophages inhibited T-cell proliferation (63). In addition, the mannose receptor (CD206), which is expressed on TAMs, has been shown to contribute to the immunosuppressive function of TAMs by binding tumor mucins such as CA125, which increases the levels of IL-10 and decreases levels of the T-cell chemo-attractant CCL3 (24). Treatment of ovarian TAMs with IFNγ is able to reduce TAM secretion of CCL18 and VEGF and switch TAMs from an immunosuppressive to an immunostimulatory phenotype (64). Inducing an M1 phenotype in ovarian TAMs, for example, via treatment with IFNγ or targeting B7-H4 may prove useful in encouraging immune destruction in patients with ovarian cancer.

As has been demonstrated in other cancers, TAMs are beginning to emerge as promoters of angiogenesis in ovarian cancer. Co-culturing of TAMs with ovarian cancer cell lines led to an increase in expression of the pro-angiogenic cytokine IL-8. The conditioned media from these co-cultures significantly increased the migration and tube formation of endothelial cells compared to conditioned media from tumor cells or TAMs alone (65), indicating interactions between tumor cells and TAMs are important for promoting angiogenesis rather than direct interactions between TAMs and endothelial cells. TAMs also promote lymphangiogenesis in ovarian cancer. An increased TAM density was found to be significantly associated with an increased lymphatic vessel density in ovarian cancer patients and TAMs were shown to promote lymphatic endothelial cell proliferation, migration, and tube formation *in vitro* (66). Further studies are required to identify the mechanisms employed by ovarian TAMs in inducing ovarian tumor angiogenesis.

## Concluding Remarks

Tumor-associated macrophages have been shown to play a key role in creating an immunosuppressive tumor microenvironment, as well as promote tumor growth, progression, metastasis, and angiogenesis. The studies mentioned in this review highlight several targets through which TAMs may be targeted therapeutically such as CSF-1, IL-6, NFκB and suggest that depletion of TAMs, or re-education to an immunostimulatory phenotype, may result in a decrease in tumor growth and spread as well as enhance response to chemotherapy. In ovarian cancer, much of the research into TAMs has so far been limited to immunohistochemical characterization in human patient samples and *in vitro* evaluation of their effects on ovarian tumor cells. Compared to other tumor types, a relatively limited number of *in vivo* studies have been performed using xenograft and syngeneic mouse models. Results from these models are potentially confounded by anti-tumorgraft reactions present in syngeneic models and biased immune interactions in xenograft models that use immunodeficient mice. The best assessment of anti-TAM treatment strategies in ovarian cancer would come from the use of spontaneous tumor models, however there are few spontaneous ovarian cancer models available (67, 68). Additionally, whether the role of TAMs varies between each of the histopathological subtypes has not been thoroughly investigated and requires further study. Nonetheless, the work summarized in this review demonstrate that TAMs represent an important component of the ovarian tumor microenvironment, and further studies will assist in evaluating this cell type as a potential therapeutic target.

## Conflict of Interest Statement

The author declares that the research was conducted in the absence of any commercial or financial relationships that could be construed as a potential conflict of interest.
